# 617. Predicting Hospitalizations for Influenza, Respiratory Syncytial Virus, and COVID-19 Using Wastewater Surveillance: A Causal Analysis

**DOI:** 10.1093/ofid/ofaf695.190

**Published:** 2026-01-11

**Authors:** Maria Akiki, Ali Hemade, Jihad Slim

**Affiliations:** University of Connecticut, Hartford, CT; Lebanese University, Beirut, Beyrouth, Lebanon; Saint Michael’s Medical Center, Newark, NJ, USA, Newark, NJ

## Abstract

**Background:**

Respiratory viral infections like influenza, RSV, and COVID-19 cause significant healthcare strain, especially during seasonal surges. Traditional surveillance often fails to provide early warnings, but wastewater surveillance offers a promising real-time alternative. This study evaluates whether wastewater viral loads predict hospitalizations and identifies the lead time between detection and hospitalization surges.

**Figure:**
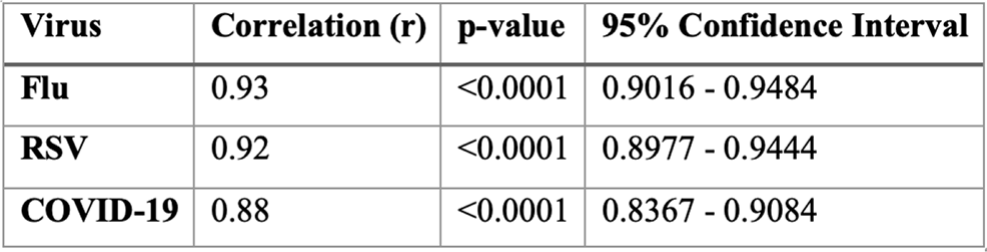
Correlation Between Wastewater Viral Load and Hospitalization Rates for Flu, RSV, and COVID-19

**Figure:**
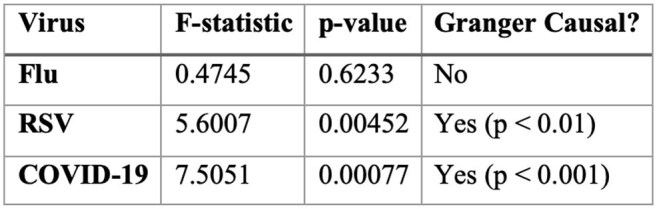
Granger Causality Analysis of Wastewater Data and Hospitalization Trends

**Methods:**

This retrospective observational study analyzed the relationship between wastewater viral loads and hospitalization trends for influenza, RSV, and COVID-19 across all 50 U.S. states from January 2021 to February 2025. Weekly viral concentrations were obtained from the National Wastewater Surveillance System and hospitalization rates from CDC reports, with datasets standardized by calendar week and no missing data. Pearson correlations, cross-correlation function analysis, granger causality testing, and distributed lag models were used to assess associations, lead times, causality, and effect sizes. Statistical significance was set at p< 0.05, and analyses were performed using R (v4.2.0). IRB approval was not required as only publicly available, de-identified data were used.

**Figure:**
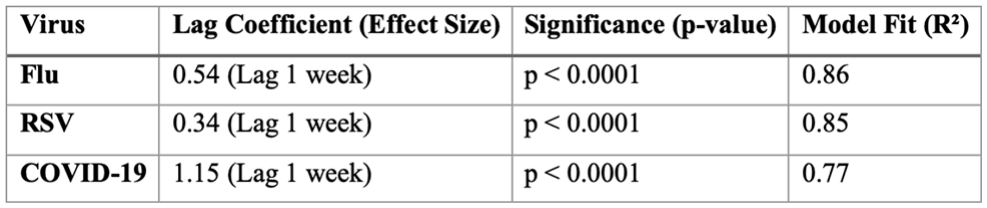
Distributed Lag Model Results: Effect of Wastewater Viral Load on Hospitalizations

**Results:**

Wastewater viral loads predicted hospitalizations, with a lead time of 2 weeks for Flu and 3 weeks for RSV and COVID-19 (p< 0.01). Correlations were observed for Flu (r = 0.93), RSV (r = 0.92), and COVID-19 (r = 0.88) (Table 1). Granger causality confirmed that RSV and COVID-19 wastewater signals predicted future hospitalizations (p< 0.01, Table 2). A one-unit rise in wastewater viral concentration corresponded to hospitalization rate increases of 0.54 for Flu, 0.34 for RSV, and 1.15 for COVID-19 (Table 3). However, Flu did not show a causal predictive relationship.

**Conclusion:**

Our study is the first to establish a causal relationship between wastewater viral loads and RSV and COVID-19 hospitalizations using Granger causality analysis, demonstrating that wastewater surveillance is a valuable tool for predicting hospitalizations weeks in advance and enabling timely public health interventions. This real-time monitoring system can improve hospital preparedness, optimize resource allocation, and guide vaccination strategies.

**Disclosures:**

All Authors: No reported disclosures

